# Influence of magnetic field on morphological structures and physiological characteristics of bEnd.3 cells cultured on polypyrrole substrates†

**DOI:** 10.1039/c9ra07180f

**Published:** 2019-12-11

**Authors:** Xue Yang, Ke Ma, Libo Yang, Yujuan Chen, Yingmin Qu, Ying Wang, Xinyue Wang, Fan Yang, Qi Sun, Zhengxun Song, Zuobin Wang

**Affiliations:** International Research Centre for Nano Handling and Manufacturing of China, Changchun University of Science and Technology Changchun 130022 China make@cust.edu.cn wangz@cust.edu.cn; School of Electronic Information Engineering, Changchun University of Science and Technology Changchun 130022 China yang_libo151@sina.com; JR3CN, IRAC, University of Bedfordshire Luton LU1 3JU UK; School of Life Sciences, Changchun University of Science and Technology Changchun 130022 China

## Abstract

This paper employs a spin-coated method to construct conductive polypyrrole (PPy) substrates which present superior properties for controlling the morphological structures and functions of bEnd.3 cells. The PPy substrates with a homogeneous particle size, uniform distribution and proper roughness show enhanced hydrophilic characteristics and improve cell adhesion to the substrates. The changes in the mechanical properties of cells and the responses to the designed substrates and magnetic field are also explored. Due to the synergistic effect between the magnetic field and the conductive PPy substrate, the cells cultured in such an environment exhibit applanate shapes with more branches and enhanced cell viability. In addition, the cells preferentially extend along the magnetic field direction. The mechanical characteristics of cells change significantly under varying magnetic intensity stimulations (5–16 mT). The satisfying effect on cells' morphology and outgrowth is acquired at the magnetic intensities of 9–10 mT and duration of 20 min, compared with other stimulated groups, while retaining cell viability. Moreover, the cells express higher adhesion up to 5.2 nN. The results suggest that the application of the PPy substrates and magnetic field is a promising candidate for the protection of neurovascular units and treatment of neurological diseases.

## Abstract 2

The combination of magnetic stimulation and polypyrrole (PPy) substrates regulate the bEnd.3 cells mechanical and physical characterizations.

## Graphical abstract

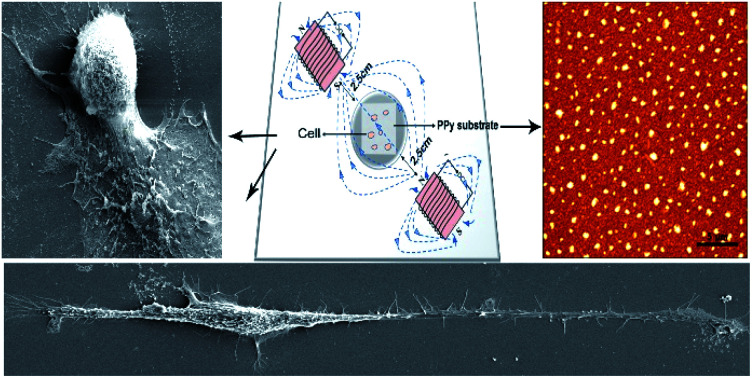

## 1. Introduction

Polypyrrole (PPy), known as the ‘organic metal’, has attracted scientists' attention for its high electrical conductivity, good chemical stability and plasticity.^1,2^ Notably, PPy exhibits good biocompatibility which allows further research into its biological applications, such as cell cultures, nerve regeneration, biosensors and biochips.^3,4^ Recently, PPy films have been extensively developed as functional materials used especially for electrophysiological applications. Previous studies have different approaches toward conductive PPy films such as the electrochemical synthesis, emulsion polymerization, oxidative polymerization, interfacial polymerization and template method.^5,6^ However, the PPy films obtained by the methods exhibit uneven surfaces and non-uniform PPy particle distributions.

Recently, some reports have demonstrated that PPy films derived from different fabrication methods significantly affected the viability of cells.^7,8^ PPy films were usually used as the extracellular environments, thus the surface roughness and PPy particle uniformity were two critical factors for cell growth. Lin *et al.* showed that nanoparticle sizes and surface properties played a key role in determining their *in vivo* fate.^9^ The non-uniform distribution of PPy particles on the membrane surface caused the heterogeneous stimulation and poor polymer–cell interaction.^10,11^ Therefore, an improvement approach was needed to obtain PPy films with more uniform PPy particle density and flatness.

In addition, most reports have described the effects of PPy films on cell growth using the tools such as optical microscope (OM)^12^ and scanning electron microscope (SEM), transmission electron microscope (TEM).^13^ Since much work has focused on how PPy films influence the cell survival, little has been reported on how PPy films change cell physical characteristics. Furthermore, quantitative examinations of the effect of cells induced by substrates are critical to promote the understanding of associated cell response mechanism. Therefore, the analytical method is noticeably essential to explore the physical characteristics of living cells on the PPy film in various conditions. To investigate the effects, atomic force microscope (AFM) has been proved to be a versatile technique and tool to monitor cell–substrate interactions quantitatively and in real time.^14^ Similarly, AFM has become one of the most popular tools for surface imaging and measuring of different materials, especially for characterizing the size distribution of nanoparticles and surface roughness.

Magnetic field stimulation is a safe and noninvasive physical therapy to promote and align nerve regeneration for nerve injury.^15^ Xu and Hughes *et al.* investigated a PPy nerve conduit, which had great potential for nerve tissue regeneration under the electrical stimulation, and the neurite length also extended.^7,16^ Che *et al.* found that magnetic simulations could affect the behavior of neural cells, promoting their proliferation and adhesion.^17^ In spite of intense research, some reports have shown no change or opposite results in cellular responses after the same stimulation.^18,19^ A significant problem is that the strength and duration of magnetic stimulations are hard to be determined. Furthermore, most cellular changes induced by magnetic stimulation remain on the observation rather than the accurate measurement. It is desirable to further explore the magnetic effect on the physical change of living cells cultured on substrates.

## 2. Experimental section

### 2.1 Materials and instruments

Pyrrole was purchased from Aladdin Co. (China). Ammonium persulfate (APS) and dimethylsulphoxide (DMSO) were purchased from Guangfu Co. (China). Sodium dodecyl benzene sulfonate (SDBS) was purchased from Xilong Science Co. (3009647, China). DMEM medium, trypsin, phosphate buffer saline (PBS) and fetal bovine serum (FBS) were purchased from Gibco (USA). Mouse brain microvascular endothelial cells (bEnd.3) were purchased from ATCC Co. (ATCC® CRL-2299, USA). Cell proliferation kit I (MTT, 11465007001) was purchased from Sigma-Aldrich.

bEnd.3 cells were incubated in a cell culture incubator (Ip610, Yamato Scientific Co. Ltd., Japan). The microscope images were recorded by an inverted optical microscope (DS-Ri2, Nikon, Japan). The images were recorded by an AFM (NanoWizard®3, JPK Instruments AG, Germany) with a MLCT probe (0.55 μm thickness, 20 nm curve radius, 22 kHz resonance frequency, 0.03 N m^−1^ spring constant, Bruker, Germany). The conductivity of PPy substrates were measured by AFM in the conductive mode. (NanoWizard®3, JPK Instruments AG, Germany) with an ACCESS-EFM-10probe (2.8 μm thickness, 25 nm curve radius, 36–98 kHz resonance frequency, 0.8–0.9 N m^−1^ spring constant, Bruker, Germany). The magnetic field generation device was constructed with a DC power supply (DF1731SL3A, Ningbo Zhongce Electronics Co., China). The magnetic induction was measured with Gaussmeter (TD208, Hengtong Magnetoelectricity Co., China). The PPy films were fabricated by spinning with a spin coater (KW-4A, Siyouyen Co., China).

### 2.2 PPy substrate fabrication

The purified pyrrole monomer was achieved as colorless transparent liquid by vacuum distillation. Different amounts of APS (0.068 g or 0.136 g) and SDBS (0.05 g) were diluted into 10 mL water to obtain a mixture. 50 μL pyrrole monomer was first dissolved in 5 mL DMSO, then poured into the above prepared mixture. After 6–48 h reaction in a sealed brown bottle at room temperature, the PPy solution was achieved. Each time, the PPy solution (20 μL) was spun on a cover glass (20 mm × 20 mm) for 1 min using a spin coater at 1000 rpm. The total spin number was from 2 to 6 times. Then the cover glasses were dried in a vacuum drying oven for 4 h at 80 °C to have the PPy substrates. Finally, the PPy substrates were located in an autoclave for 30 min sterilization. The sterilized PPy substrates can be put into the culture cover glass for cell culture.

### 2.3 Cell culture

The cells were cultured in a T25 cm^2^ culture flask using the DMEM medium supplied with 10% fetal bovine serum (FBS) and 1% antibiotic. The culture flasks were incubated in a humidified atmosphere with 5% CO_2_ at 37 °C in a cell culture incubator (Sanyo, Japan). The cells were subcultured when they reached 80% confluence, and they were washed twice using DPBS without calcium and magnesium followed by detaching the cells from the flask using 1 mL trypsin for 2 min at 37 °C in the cell culture incubator. Then 2 mL fresh culture medium was added to neutralize trypsin followed by transferring the mixture into a centrifuge tube and carry out the centrifuge for 5 min at 1000 rpm. The supernatant was discarded and the cell pellets were diluted using fresh media. After that the diluted cells were dispersed on 38 mm culture cover glass (with or without the PPy substrates) for another 24 h incubation before use.

### 2.4 Magnetic field generation device

The magnetic field generation device was used to stimulate the cells consisting of a direct-current (DC) power generator, coil and gaussmeter. Different DC currents (0.35, 0.45 and 0.55 A) were applied to well-distributed coils (ring shape, copper wires, 0.5 mm diameter, 500 turns). The experimental device is shown in Fig. S1.† Two coils were diagonally placed, and the culture cover glass was equidistantly located between the two coils. The horizontal angle of the magnetic field was 45°. After the magnetic stimulation, the cells were directly measured by AFM and inverted optical microscope.

### 2.5 PPy substrates analysis process

The surface morphology and the conductivity were tested in five selected distinct regions on a PPy substrate by AFM with the scan range of 80 × 80 μm^2^. Then, the data of five selected regions were averaged to represent the value of each PPy substrate.

Before the cell incubation, the surface physical characteristics of the sterilized PPy substrates were tested in five different regions using AFM in air. After incubating cells on PPy substrates in a cover glass, the physical characteristics of cells were measured 20 times by AFM in culture. During the experiment, five cells in a cover glass were scanned to obtain their images. Then five regions were selected for each cell to analyze the characteristic parameters (adhesion and Young's modulus) by image processing software.

After the different magnetic treatments using the magnetic field generation device, the physical characteristics of cells on PPy substrates were tested 10 times with the inverted optical microscope and AFM in culture. Then the five regions were selected for each cell to analyze their characteristic parameters (adhesion and Young's modulus) by image processing software.

### 2.6 Cytotoxicity analysis process

The cells were seeded on different PPy substrates in a 6-well plate at a density of 1.5 × 105 cells per well, and were incubated in the cell culture incubator for 24 h at 37 °C. Then the Cell Proliferation Kit I (MTT) assay was immediately added to investigate the cell viability under the influence of different PPy substrates.

### 2.7 Image J analysis of cell branch length

The cell branch measurement is the straight-line distance from the longest branch to the cell body. All the cells that cultured on the PPy substrate or on the cover glass were measured by an inverted optical microscope. The branch length of total 60 cells from each group were analyzed by Image J software.

## 3. Results and discussion

### 3.1 Characterization of PPy substrates

Ammonium persulfate as a catalyst played an important role in increasing the reaction rate of the PPy synthesis. As shown in Fig. S2,† the mixed solution color went dark with the reaction time (6–48 h) in the presence of 0.068 g ammonium persulfate, due to the generation of PPy particles. In comparison, the mixed solution showed a black color after 36 h reaction in the presence of 0.136 g ammonium persulfate, and it had almost no PPy particle precipitation as the reaction time increased due to the completed reaction of the mixed solution. Therefore, the two PPy solutions both reacted completely for the fabrication of PPy substrates after 48 h.

Six types of PPy substrates were produced with different PPy solutions and spin times. As shown in Table 1, 20 μL PPy solution was used for spin coating at each time, then next spin began after 2 min standing. The total spin number was 2, 4 or 6 times. In the presence of 0.068 g catalyst (group 1), the PPy substrates were labelled as 1.1, 1.2 and 1.3. The PPy substrates with 0.136 g catalyst (group 2) were named 2.1, 2.2 and 2.3.

**Table 1:** Groups of polypyrrole (PPy) substrates

Catalyst (g)	PPy substrate name		
	Spin number		
	2 times	4 times	6 times
Group 1 0.068	1.1	1.2	1.3
Group 2 0.136	2.1	2.2	2.3

**Fig. 1:**
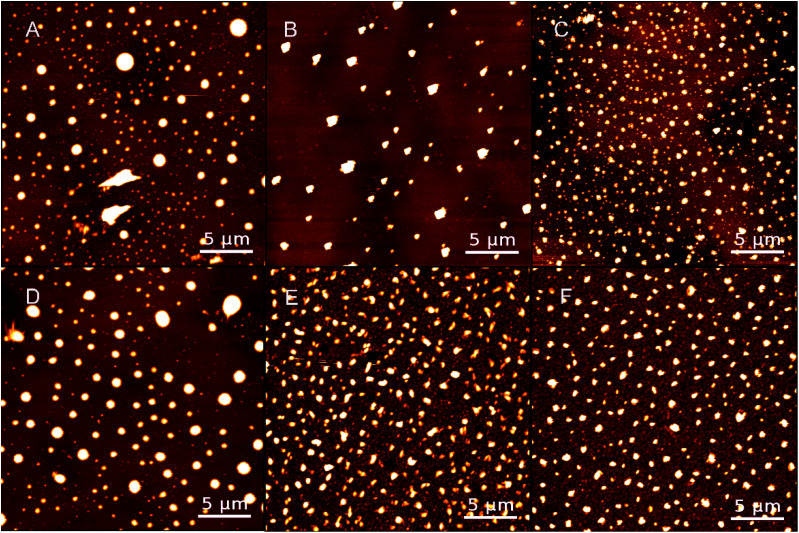
AFM images of PPy substrates 1.1, 1.2 and 1.3 (A–C) and PPy substrate 2.1, 2.2, and 2.3 (D–F); scale bars: 5 μm.

The surface morphological images of PPy substrates were obtained by AFM in Fig. 1, which showed the distributions of PPy particles. Meanwhile, the SEM images of PPy substrates were shown in Fig. S3.† Both AFM or SEM images of PPy substrate, the diameters of PPy particles for both groups (group 1 and group 2) had a gradual decline with the increase of spin number, and the particle distribution became more uniform (Fig. S4†), along with higher particle density and film thickness (Table S1†). The PPy substrate 2.3 presented better uniformity of PPy particles.

Recent publications reported that DMSO as an organic solvent could solve the insoluble and infusible problems in synthesizing conductive PPy. Meanwhile, partially avoided the collision and aggregation of particles based on its solvent effect.^20,21^ Thus, with more spin number in fabricating PPy substrates, more DMSO solvents were contained, resulting in a better uniform distribution of particles with smaller sizes. Ammonium persulfate (APS), playing a synergistic role in adjusting the morphology of PPy molecules as a surfactant. Comparing with the two groups, the range of particle size and film thickness of group 2 were smaller than those of group 1, indicating that adding more catalysts in the PPy fabrication would acquire smaller sizes of synthetic PPy and generate PPy particles. In addition, a few snowflake structures were found on the edge of PPy substrates (Fig. S5†), which was probably due to the co-crystallization of SDBS and PPy particles.^22,23^

The surface characteristics of substrates were critical to provide a suitable micro-environment for cell growth.^24,25^ Therefore, the roughness and hydrophilicity of PPy substrates were analyzed to obtain the optimal condition for cell culture. All the morphological images in Fig. 1 clearly showed that PPy substrates had an interconnected surface structure through the multilayer spin coating. The roughness of PPy substrates declined with the increasing number of spin coating (Table S2†), indicated that the interconnected multilayer structure could smooth the PPy substrate. The roughness values of PPy substrates 1.3 and 2.3 were slightly higher than that on the cover glass surface (control group), meanwhile, they were the closest roughness value to the cover glass surface. Another factor of examination was the surface hydrophilicity, as shown in Table S2.† The hydrophilicity of PPy substrates had an irregular change with the increasing number of spin coating.

The PPy substrate 2.2 presented better hydrophilicity with a contact angle of 15 ± 5°, and PPy substrate 1.2 had worse hydrophilicity with a contact angle of 40 ± 5°. It was well known that the good substrate for cell culture was amphipathic (not extremely hydrophilic).^2,26^ Thus, the contact angles of all PPy substrates (from 15 ± 5° to 40 ± 5°) may have different effects on cell growth compared with the control group (35 ± 5°). According to the report, a coating was applied to improve a specific function or enhance surface properties.^27^ Therefore, in addition to the changes in the PPy substrate wettability, the physical and chemical properties of material surface also affected the roughness, morphology, particle size, film thickness, and the stability of the PPy substrate. In the combination of roughness and hydrophilicity, PPy substrate 2.3 would be more desirable for cell culture, which was similar to the control group.

**Fig. 2:**
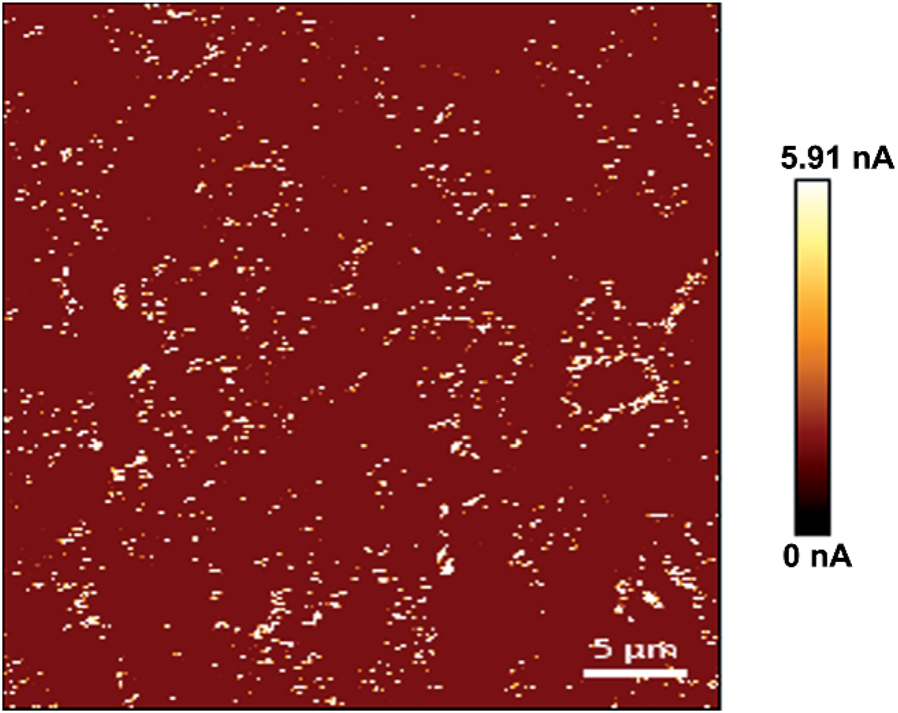
The conductive image of PPy substrate 2.3.

Similarly, the electrical characteristics of PPy substrates were analyzed by AFM in the conductive mode. Based on the measurement results, the conductivity of PPy substrates varied from 1.61 to 5.91 nA and gradually enhanced with the increase of spin coating, which had no negative influence on the cell culture. As shown in Fig. 2, the conductive image of PPy substrate 2.3 was showed and the current intensity reached the maximum value of 5.91 nA. The white region indicated high conductivity in PPy substrate 2.3. In addition, we quantified the conductivity of all white regions, and the relative current intensity distribution was shown in Fig. S6.† It was observed that the current intensity was mainly in the range of 2–4 nA.

### 3.2 Biological analysis of cells on PPy substrate

Microscopic imaging observations and cell viability detection were performed to investigate the physical properties of cells cultured on PPy substrates. The survival rate of cells was measured using the MTT assay (Fig. S7†). The cell viability gradually increased in group 1 (PPy substrates 1.1–1.3) and group 2 (PPy substrates 2.1–2.3), and the cells in group 2 presented higher cell viability than that in group 1. Especially the cells on PPy substrate 2.3 had the highest cell viability at about 98.9%, indicating that PPy substrate 2.3 had better material surface properties for cell culture.

The surface morphology with micro- and nano-scale features might have beneficial effects on the cell–substrate interaction, which significantly influenced the adsorption of proteins and thus, it determined also the adhesion, spreading and growth of the cells *in vitro*.^28^ In addition, there were longer cell branches on the PPy substrates (group 2) compared to that on the cover glass surface (control group) (Fig. S8a†). The branch length distribution of bEnd.3 cells was shown in Fig. S8b and c.† The average branch length for the control group was about 38.20 μm, and it turned into 36.93 μm, 31.77 μm, 37.28 μm, 40.82 μm, 38.92 μm and 63.54 μm for the 1.1, 1.2, 1.3, 2.1, 2.2 and 2.3 substrates, respectively. Compared with the control group, the average branch length of bEnd.3 cells in group 1 was decreased, but it was extended in group 2. The bEnd.3 cells on PPy substrate 2.3 had the longest branch with the average elongation of 1.66-fold which could accelerate the communication between cells. It was probably due to that an increase of cell–substrate adhesion could induce the branching outgrowth.^29,30^

As mentioned above, PPy substrates 2.1–2.3 (group 2) mimicked a better micro-environment for cell culture, thus, they were selected to study the cell properties including cell adhesion and Young's modulus (Fig. S9†) by AFM. All the cells on PPy substrates had higher cell adhesion compared to the control group (2.3 > 2.2 > 2.1 > control), which led to the extension of cell branches. Especially, the adhesion of cells on PPy substrate 2.3 presented a slight increase at about 4.09 nN (Fig. S9a†). Thus, the cells on PPy substrate 2.3 substrate might have the longest branch. Fig. S10† shows the cell branches on PPy substrate 2.3 and the control group, respectively. The cell branch extended from 29.03 μm (control group) to 74.44 μm (PPy substrate 2.3), along with the extending of 2.56-fold. According to previous studies, the high adhesion value of cells could promote the secretion and adsorption of more proteins and enhance cell branching out-growth under electrical stimulation by PPy substrate.^31,32^ The Young's modulus of cells also showed that the control group had the highest Young's modulus at about 5.14 kPa (control > 2.2 > 2.3 > 2.1, Fig. S9b†), and the cell Young's modulus for PPy substrates 2.2 and 2.3 were similar (3.70 kPa and 3.58 kPa, respectively). Fig. S11† shows the numerical range of cell adhesion and Young's modulus. Detailed mechanical parameters have become an essential factor to estimate the physiological status of cells, which directly affects the cytoskeleton and membrane elasticity. It was reported that the cell elasticity was implicated in many progressive diseases, meanwhile, the enhancement of cell adhesion was an important criterion when designing biomaterials for neural tissue engineering.^33,34^ In addition, dorsal root ganglion (DRG) neurons and glial cells, primary cortical and spinal cord neurons were reported to grow well with elastic moduli on the order of a few hundred Pa,^35^ and bEnd.3 cells were part of the neurovascular unit which the values of Young's modulus were served as reference data.

The optimal surface of biocompatible materials should possess suitable parameters in promoting the cell growth and at the same time with a “smart” response of the surface.^36^ In context, PPy substrate 2.3 would be more desirable for cell culture due to the high cell viability, long cell branch, high cell adhesion and proper Young's modulus, and it will be discussed in the next section.

### 3.3 Magnetic effect of PPy substrate on cells

Magnetic stimulation has a two-way regulation of the promotion and inhibition of cell survival.^37,38^ The inverted optical microscope was used to investigate the magnetic effects on cells with different magnetic intensities and stimulation durations. Firstly, the morphology of bEnd.3 cells was elongated and somewhat like fibroblasts with no magnetic stimulation in PPy substrate or control group (Fig. S12†). As shown in Fig. S13,† the cells on PPy substrate 2.3 had no change under the 10 min magnetic stimulation with different magnetic intensities, while the longer stimulation duration (15–20 min) obviously changed the cell physiological status. Nearly 80% cells cultured on PPy substrate 2.3 presented an oriented growth compared to the horizontal direction (45–90°) after 15 min stimulation due to the effect of magnetic field.

The growth orientation of cells was changed to 45–60° after 20 min stimulation, which was approximative along the direction of the magnetic field (45°), and the morphology of cells obviously turned into the applanate shape. Further longer stimulation time (30 min) led to the death of most bEnd.3 cells. As shown in Fig. S14,† the viability of cells decreased significantly after the magnetic stimulation for 30 min and 40 min which indicated a long time stimulation inhibited the cell survival. Besides, the cells on the cover glass surface (control group) had a similar cell growth orientation but no apparent cell morphological changes under the same magnetic stimulation. It indicated that the orientation was induced by the magnetic effect,^39–41^ but the cell morphological change was probable due to the generation of magnetic field, which induced an electromagnetic effect on PPy substrates and stimulated the cells.^42,43^ Another interesting observation was the cell density-dependent effect under the magnetic stimulation. As shown in Fig. S15,† the cells with a high cell density on PPy substrate 2.3 displayed more degree of orientation and tightly interconnected, while the orientation was partially inhibited in the low cell density region. It was speculated that the cells with the high density might secrete more extracellular matrix (ECM) for inducing neurotransmitter release, which led to the orientation of cells and accelerated intercellular communications through the magnetic effect.^44,45^

**Fig. 3:**
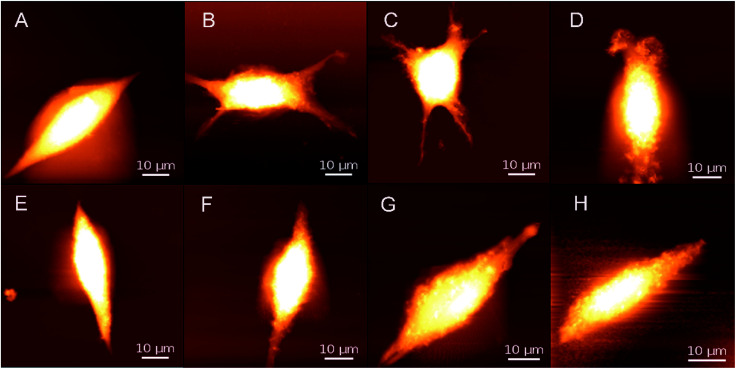
AFM images of cells on PPy substrate 2.3 (A–D) and the cover glass surface (control group) (E–H) after 20 min magnetic stimulation with different magnetic intensities; DC currents and magnetic intensities: (A and E) 0 A and 0 mT, (B and F) 0.35 A and 5–6 mT, (C and G) 0.45 A and 9–10 mT, and (D and H) 0.55 A and 15–16 mT.

Next, the physical characteristics of cells based on PPy substrate 2.3 after 20 min magnetic stimulation were studied with different magnetic intensities (0 mT, 5–6 mT, 9–10 mT, and 15–16 mT). Fig. 3 shows the AFM images of cells on PPy substrate 2.3 and the cover glass surface (control group). Without the magnetic stimulation, the cell surface was smooth, after the stimulation with the magnetic intensities of 5–6 mT or 9–10 mT, the cells turned into the applanate shape with more cell branches, which benefited the cell tightly connectivity and signal transmission between the cells.^18^ In comparison, the cells on the cover glass surface (control group) had few morphological changes under the stimulation. To further testify the increased amounts of cell branches, the magnetic intensities of 9–10 mT were chosen to stimulate the cells on PPy substrate 2.3 for 20 min. Compared to the unstimulated groups, the bEnd.3 cells cultured in the magnetic field showed longer extensions, and particularly many cells preferentially generated more branches from their bodies after the stimulation (Fig. S16†). It demonstrated that the magnetic stimulation could increase cell branches on PPy substrate 2.3. When the magnetic intensities were increased to 15–16 mT, the cells gradually disintegrated (Fig. 3). The apoptosis trend of the control group was more distinct than that of PPy substrate 2.3, and the results from the images led to the conclusion that the magnetic intensities of 15–16 mT were excessive and adverse for cell culture. Thus, 5–6 mT and 9–10 mT would be appropriate for cell culture under the magnetic stimulation on PPy substrates.

**Fig. 4:**
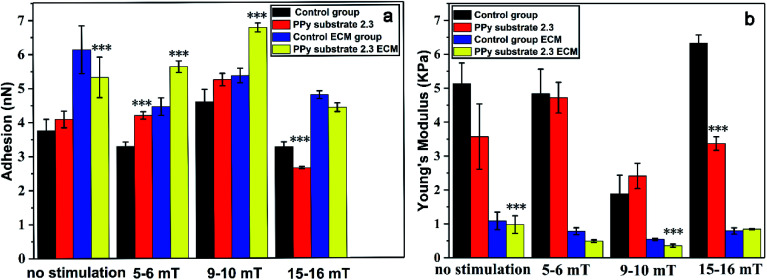
(a) The adhesion and (b) Young's modulus of cells and ECM on PPy substrate 2.3 and the cover glass surface (control group) after 20 min magnetic stimulation with different magnetic intensities: 5–6, 9–10 and 15–16 mT. All the results are expressed as the mean values and standard errors. The cell number is 5 for each group (the results represent means ± s.e.m. * represents *p* ≤ 0.05, ** represents *p* ≤ 0.01 and *** represents *p* ≤ 0.001 compared to the control group).

In order to determine the effects of the magnetic stimulation on the cells cultured on PPy substrates, the adhesion, Young's modulus of cells and the relative ECM before and after the magnetic stimulation are shown in Fig. 4. The adhesion of cells was gradually increased with the increase of magnetic intensities (5–6 mT, 9–10 mT), and the opposite result presented a rapid decline with the magnetic intensities of 15–16 mT. It was inferred that the high intensity of magnetic field caused the adhesion lower than that without the stimulation, which was not suitable for cell growth. At the same time, the adhesion tendency of ECM was in a good agreement with the trend of adhesion changes. The result was interpreted as that the magnetic stimulation (magnetic intensities of 5–6 mT and 9–10 mT) could increase the adhesion of ECM due to the more secretions of proteins.^46^ But the stimulation with a higher magnetic intensity destroyed the growth of cells which led to fewer secretions of proteins and decreased the adhesion of ECM.

However, the Young's modulus of cells had different presentations with the adhesion and ECM adhesion. Compared to the unstimulated cells, the Young's modulus of cells in the control group had few changes under the magnetic stimulation at the intensities of 5–6 mT, but increased on PPy substrate 2.3, which might affect the secretion of proteins by the cytoskeleton tension change.^47,48^ When the magnetic intensities were increased to 9–10 mT, the Young's modulus of cells had a sudden decline and significantly increased with the magnetic intensities of 15–16 mT. The Young's modulus of ECM was decreased at lower magnetic intensities of 5–6 mT and 9–10 mT due to the more secretions of proteins, but increased with the magnetic intensities of 9–10 mT. Probably, the higher magnetic intensity could cause the death of cells. The results indicated that the apoptosis process induced by the magnetic intensities of 15–16 mT could increase Young's modulus of both cells and ECM. The substrate–cell interactions and physical stimuli had clear impacts on cell behaviors and functions. Therefore, the cells cultured on PPy substrate 2.3 under the magnetic stimulation of 9–10 mT had its high adhesion and low Young's modulus, which had an optimal environment for cells culture and intercellular communications.

Finally, the cell viability was assessed by MTT analysis. As shown in Fig. S17,† the cell viability was almost 100% for the cells on PPy substrate 2.3 and the control group with no stimulation. After the stimulation at the magnetic intensities of 5–6 mT, the cell viability was slightly increased due to the magnetic effect, but the cell viability on PPy substrate 2.3 was lower than that in the control group. A further increase of magnetic intensities (9–10 mT and 15–16 mT) led to the decrease in cell viability, and the cell viability on PPy substrate 2.3 was higher than that in the control group. In this context, PPy substrate 2.3 was desirable to improve the adhesion and proliferation of cells which provided an environment with appropriate physical and chemical properties.

## 4. Conclusion

In this work, the PPy substrates were fabricated for cell culture by spin-coated method. The PPy substrate presented different characteristics with the homogeneous particle size, uniform distribution, various roughness and enhanced hydrophilic. The optimal PPy substrate for cell culture extended the branch length with the average elongation of 1.66-fold without cytotoxicity. The potential of combining AFM with complementary techniques including inverted optical microscopy, microplate spectrophotometry and DC power supply stimulation was outlined to characterize the intracellular distribution of mechanical responses within biological systems and to track their morphology and functional state. The magnetic stimulation combined PPy substrates regulated the cell growth direction that showed a density-dependent effect. In addition, the cell on the PPy substrate turned to be applanate with more branches, which could benefit the cells connectivity and intercellular communications. The positive magnetic effect was obtained at the magnetic intensities of 9–10 mT after 20 min stimulation, resulting in the high adhesion and low Young's modulus of both cells and ECM. These results suggest that the conductive PPy substrate and magnetic field plays an important role in protecting neurovascular unit and improving damaged neurons, which may provide a basis for the treatment of neurological diseases.

## Conflicts of interest

There are no conflicts of interest to declare.

## Supplementary Material

RA-009-C9RA07180F-s001

## References

[cit1] Khomenko V., Frackowiak E., Béguin F. (2005). Electrochim. Acta.

[cit2] Upadhyay J., Kumar A., Gupta K., Mandal M. (2015). Carbohydr. Polym..

[cit3] Snook G. A., Kao P., Best A. S. (2011). J. Power Sources.

[cit4] Bendrea A. D., Cianga L., Cianga I. (2011). J. Biomater. Appl..

[cit5] Liu Y. C., Chung K. C. (2003). Synth. Met..

[cit6] Mahmoudian M. R., Basirun W. J., Alias Y. (2011). Prog. Org. Coat..

[cit7] Xu H. X., Holzwarth J. M., Yan Y. H., Xu P. H., Zheng H., Yin Y. X., Li S. P., Ma P. X. (2014). Biomaterials.

[cit8] Shi G. X., Rouabhia M., Wang Z., Dao L. H., Zhang Z. (2004). Biomaterials.

[cit9] Lin X. Y., Zhang N. N., Yan P., Hu H., Xu F. J. (2015). Acta Biomater..

[cit10] Shi G., Rouabhia M., Meng S. Y., Zhang Z. (2010). J. Biomed. Mater. Res., Part A.

[cit11] Tian H. Y., Tang Z. H., Zhuang X. L., Chen X. S., Jing X. B. (2012). Prog. Polym. Sci..

[cit12] Muller D., Silva J. P., Rambo C. R., Barra G. M. O., Dourado F. (2013). J. Biomater. Sci., Polym. Ed..

[cit13] Song J. L., Sun B. B., Liu S., Chen W., Zhang Y. Z., Wang C. Y., Mo X. M., Che J. Y., Ouyang Y. M., Yuan W. E., Fan C. Y. (2016). Front. Mol. Neurosci..

[cit14] Krieg M., Fläschner G., Alsteens D., Gaub B. M., Roos W. H., Wuite G. J. L., Gaub H. E., Gerber C., Dufrêne Y. F., Müller D. J. (2018). Nat. Rev. Phys..

[cit15] Schimmelpfeng J., Weibezahn K. F., Dertinger H. (2010). Bioelectromagnetics.

[cit16] Hughes G. A. (2005). Nanomedicine.

[cit17] Che X. C., Boldrey J., Zhong X. J., Unnikandam-Veettil S., Schneider I. C., Jiles D., Que L. (2018). ACS Appl. Mater. Interfaces.

[cit18] Stem S., Rotem A., Burnishev Y., Weinreb E., Moses E. (2017). J. Visualized Exp..

[cit19] Grehl S., Viola H. M., Fuller-Carter P. I., Carter K. W., Dunlop S. A., Hool L. C., Sherrard R. M., Rodger J. (2015). Brain Stimulation.

[cit20] Gurtovenko A. A., Patra M., Karttunen M., Vattulainen I. (2004). Biophys. J..

[cit21] Pal R., Mamidi M. K., Das A. K., Bhonde R. (2012). Arch. Toxicol..

[cit22] Zhao B. B., Nan Z. D. (2012). Mater. Sci. Eng., C.

[cit23] Zeng J. W., Huang Z. B., Yin G. F. (2013). Mater. Sci..

[cit24] Hou S. P., Ji M., Fan Y. W., Lu Q., Yang H., Xu Q. Y., Cui F. J. (2003). Chin. J. Neurosci..

[cit25] Wang X. M., He J., Wang Y., Cui F. Z. (2012). Interface Focus.

[cit26] Sheng Y. J., Jiang S., Tsao H. K. (2007). J. Chem. Phys..

[cit27] Barb R. A., Hrelescu C., Dong L., Heitz J., Siegel J., Slepička P., Vosmanská V., Švorčík V., Magnus B., Marksteiner R., Schernthaner M., Groschner K. (2014). Appl. Phys. A: Mater. Sci. Process..

[cit28] SlepičKa P., Siegel J., Lyutakov O., Kasálková N. S., Kasálková Z., Bačáková L., Švorčík V. (2017). Biotechnol. Adv..

[cit29] Zhang J. G., Qiu K. X., Sun B. B., Fang J., Zhang K. H., Hamshary H. E., Deyab S. S. A., Mo X. M. (2014). J. Mater. Chem. B.

[cit30] Say F., Altunkaynak B. Z., Coskun S., Deniz O. G., Yildiz C., Altum G., Kaplan A. A., Kayin S. E., Piskin A. (2016). J. Chem. Neuroanat..

[cit31] Forciniti L., Ybarra J., Schmidt C. E. (2014). Acta Biomater..

[cit32] Zhou X., Yang X., Huanginx Z., Yin G., Pu X., Jin J. (2017). Colloids Surf., B.

[cit33] Codan B., Martineli V., Mestroni L., Sbaizero O. (2013). Mater. Sci. Eng., C.

[cit34] Yu L. M. Y., Leipzig N. D., Shoichet M. S. (2008). Mater. Today.

[cit35] Kim S., Im W. S., Kang L., Lee S. T., Chu K., Kim B. I. (2008). J. Neurosci. Methods.

[cit36] Subbiahdoss G., Kuijer R., Grijpma D. W., Mei H. C. V. D., Busscher H. J. (2009). Acta Biomater..

[cit37] Benali A., Jörn T., Weiler E., Mix A., Petrasch-Parwez E., Girzalsky W., Eysel U. T., Erdmann R., Funke K. (2011). J. Neurosci..

[cit38] Meyer J. F., Wolf B., Gross G. W. (2009). IEEE Trans. Biomed. Eng..

[cit39] McCaig C. D., Rajnicek A. M., Song B., Zhao M. (2005). Physiol. Rev..

[cit40] Cormie P., Robinson K. R. (2007). Neurosci. Lett..

[cit41] Durgam H., Sapp S., Deister C., Khaing Z., Chang E., Luebben S., Schmidt C. E. (2010). J. Biomater. Sci., Polym. Ed..

[cit42] Chen L., Omenzetter A., Schnakenberg U., Maybeck V., Offenhäusser A., Krause H. J. (2016). Sens. Actuators, B.

[cit43] He Y., Wang S. H., Mu J., Dai L. F., Zhang Z., Sun Y. A., Shi W., Ge D. T. (2017). Mater. Sci. Eng., C.

[cit44] Zhang Z., Rouabhia M., Wang Z. X., Roberge C., Shi G. X., Roche P., Li J. M., Dao L. H. (2007). Artif. Organs.

[cit45] Adel M., Zahmatkeshan M., Johari B., Kharmin S., Mehdizadeh M., Bolouri B., Rezayat S. M. (2017). Microelectron. Eng..

[cit46] Nguyen H. T., Wei C., Chow J. K., Nguy L., Nguyen H. K., Schmidt C. E. (2013). J. Neural Eng..

[cit47] Wang H. J., Chen H., Jiang B. X., Yu S. Q., Xu X. Y. (2019). Rev. Neurosci..

[cit48] Das S., Carnicer-Lombarte A., Fawcett J. W., Bora U. (2016). Prog. Neurobiol..

