# Supplementary material for: Influence of magnetic field on morphological structures and physiological characteristics of bEnd.3 cells cultured on polypyrrole substrates
Source: RSC Adv. 2019 Dec 11;9(70):40887–94. doi: 10.1039/c9ra07180f (PMC9076427; doi:10.1039/c9ra07180f)
Supplement: RA-009-C9RA07180F-s001 [file RA-009-C9RA07180F-s001.pdf]

Electronic Supplementary Material (ESI) for RSC Advances.
This journal is © The Royal Society of Chemistry 2019

Influence of magnetic field on morphological structures and physiological characteristics of bEnd.3 cells cultured on polypyrrole substrates

Xue Yang,[a] Ke Ma,*[a] Libo Yang,*[b] Yujuan Chen,[a,d] Yingmin Qu,[a] Ying Wang,[a] Xinyue Wang,[a] Fan Yang,[a] Qi Sun,[b] Zhengxun Song[a] and Zuobin Wang*[a,c]

Supplementary Information

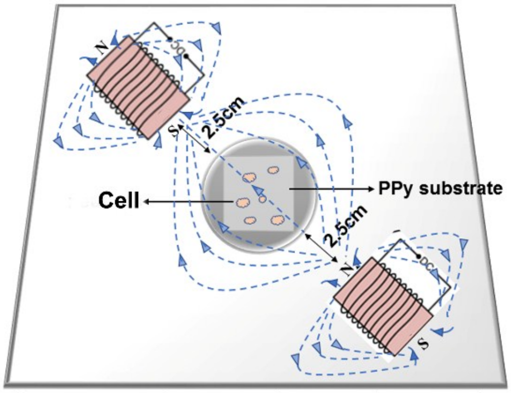

Current (A)	0.35	0.45	0.55
Magnetic intensity (mT)	5-6	9-10	15-16

Figure S1 - The experimental device for the stimulation of cells with different magnetic field intensities. DC currents and magnetic intensities: 0.35 A, 5 - 6 mT; 0.45 A, 9 - 10 mT; 0.55 A, 15 - 16 mT.

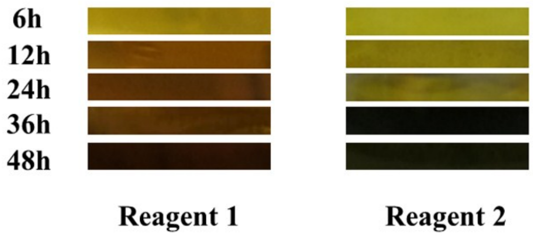

Figure S2 - The color changes of polypyrrole (PPy) solutions with different reaction time durations (6 - 48 h) in the presence of (reagent 1) 0.068 g or (reagent 2) 0.136 g ammonium persulfate.

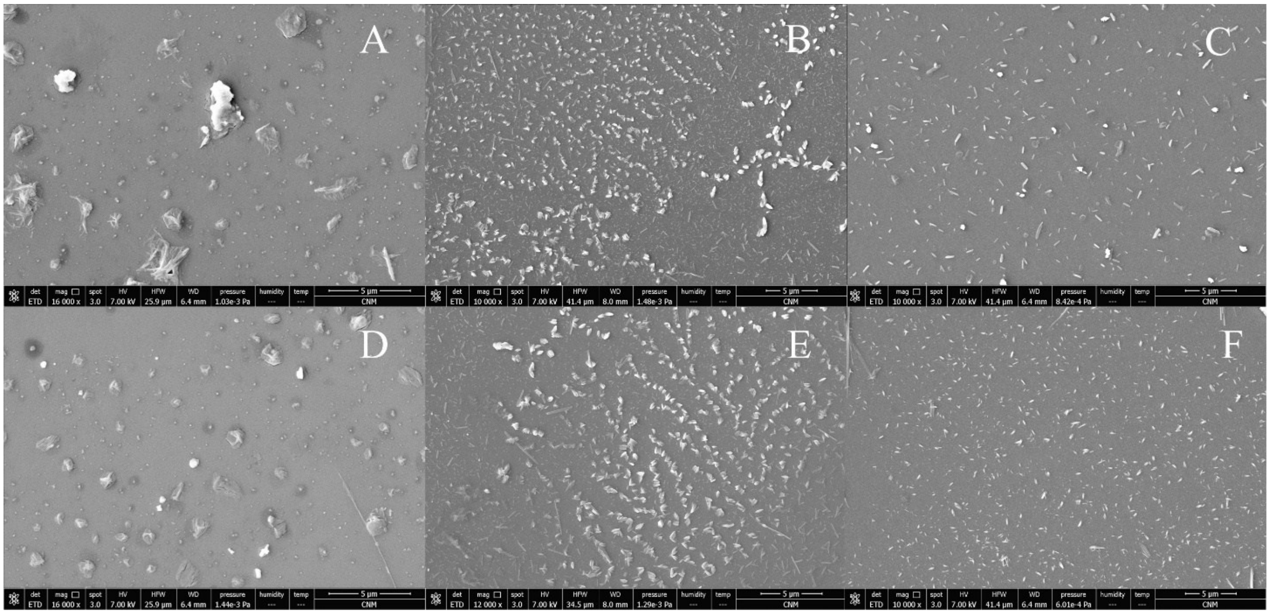

Figure S3 - SEM images of PPy substrates 1.1, 1.2 and 1.3 (A-C) and PPy substrate 2.1, 2.2 and 2.3 (D-F).

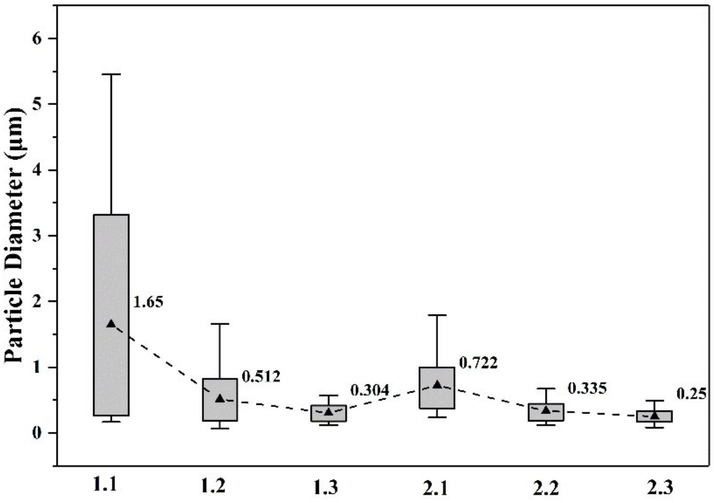

Figure S4 - The particle diameter distribution of PPy substrates 1.1, 1.2 and 1.3 (group 1) and PPy substrate 2.1, 2.2 and 2.3 (group 2). The error bars represent the standard deviation. The triangle shape and numerical value represent the average particle diameter.

Table S1. The parameters of PPy substrates.

PPy Substrate Name	1.1	1.2	1.3	2.1	2.2	2.3
Particle Size (μm)	0.34-5.80	0.26-1.70	0.18-0.45	0.51-1.85	0.23-0.67	0.26-0.46
Film Thickness (μm)	40-50	100-110	150-160	30-40	90-100	110-120

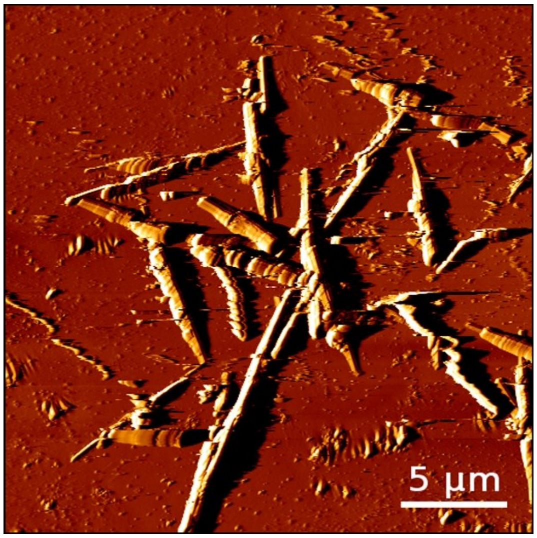

Figure S5 - AFM image of polypyrrole substrate 1.2 (crystalline domain).

Table S2. The roughness and contact angle of PPy substrates.

PPy Substrate Name	1.1	1.2	1.3	2.1	2.2	2.3	Control Group
Average Roughness (nm)	8.73	13.19	3.93	12.94	9.92	5.73	2.10
Contact Angle [°]	30 ± 5°	40 ± 5°	20 ± 5°	20 ± 5°	15 ± 5°	25 ± 5°	35 ± 5°

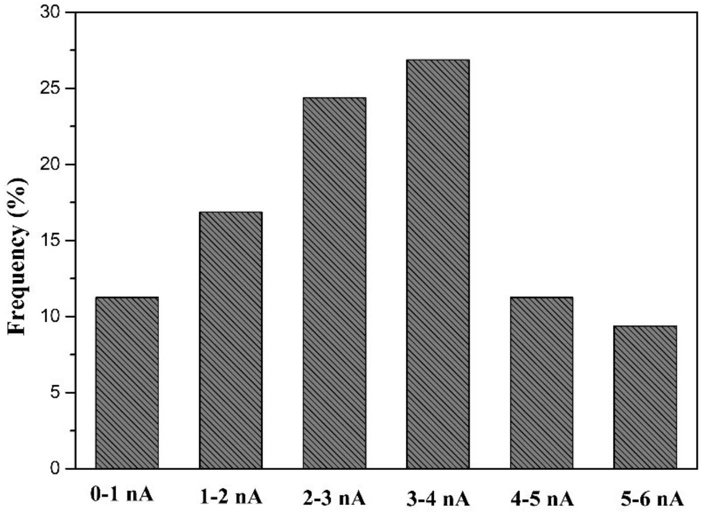

Figure S6 - The current intensity distribution of white regions in PPy substrate 2.3

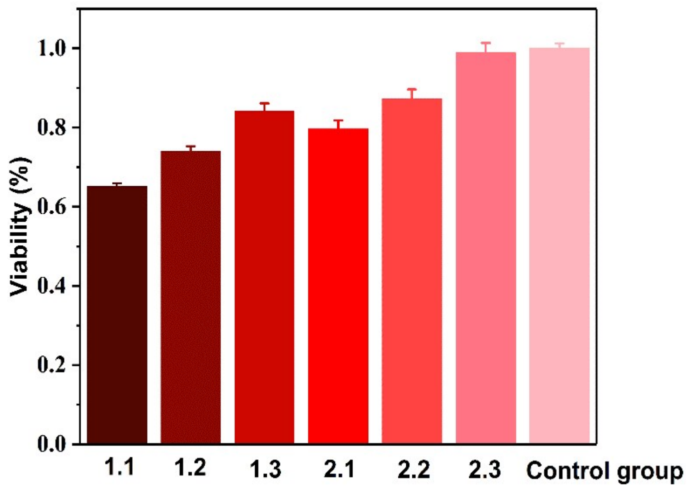

Figure S7 – Cell viability on polypyrrole substrates and control group. Error bars represent the standard deviation.

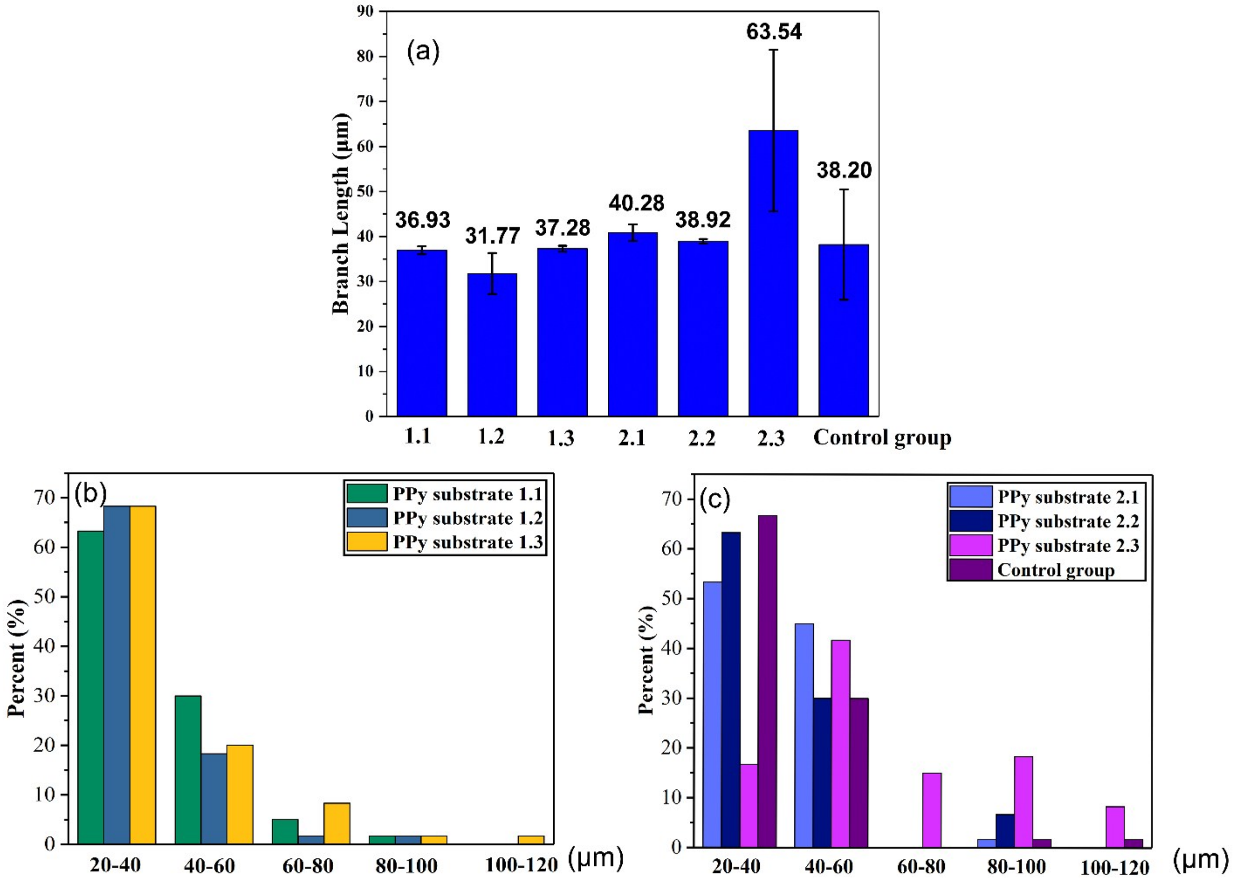

Figure S8 – The average branch length of cells in group 1 (polypyrrole substrates 1.1, 1.2 and 1.3), group 2 (polypyrrole substrates 2.1, 2.2 and 2.3) and control group; The branch length distributions of bEnd.3 cells in (b) group 1, (c) group 2 and the control group; 60 cells were analyzed for each group and then calculated by Image J processing software. The error bars represent the standard deviation.

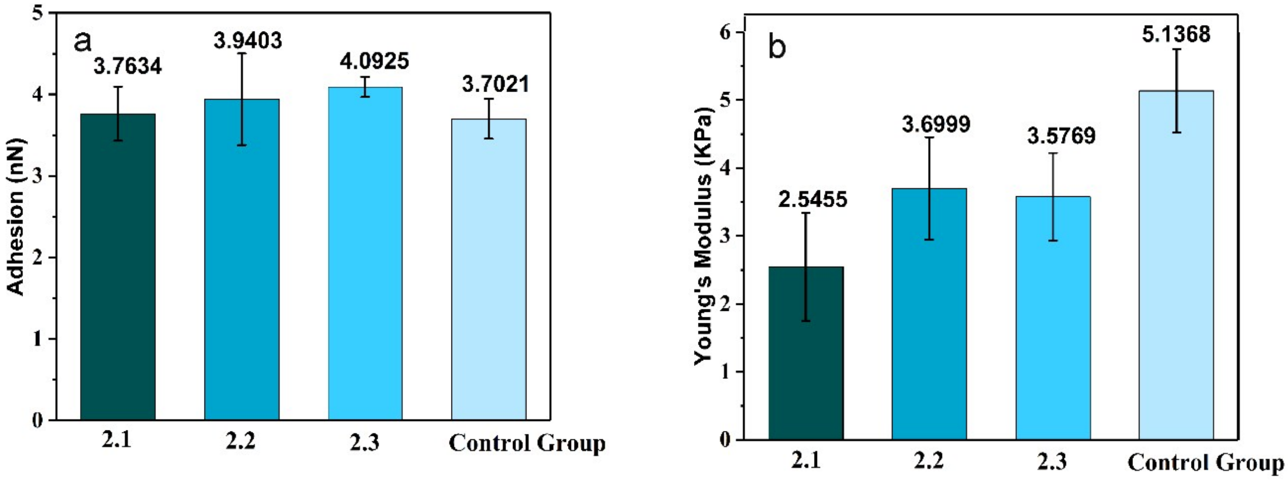

Figure S9 - (a) The adhesion and (b) Young's modulus of cells in group 2 (polypyrrole substrates 2.1, 2.2 and 2.3) or the control group; 80 cells were tested for each group and then calculated by JPK data processing software. Error bars represent the standard deviation.

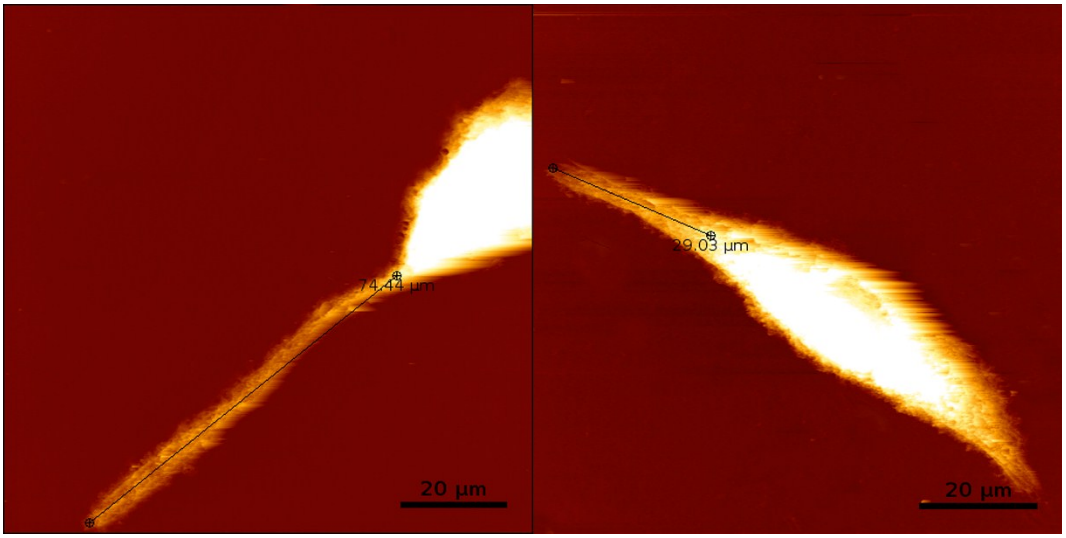

Figure S10 - AFM images of the branch length of cells (a) on polypyrrole substrate 2.3 or (b) in the control group.

Substrate Name	Range of Adhesion (nN)	Range of Young's Modulus (Kpa)
**PPy Substrate 2.1**	**3.0-4.5**	**1.5-4.5**
**PPy Substrate 2.2**	**2.4-5.0**	**2.0-6.0**
**PPy Substrate 2.3**	**3.5-4.3**	**2.0-5.0**
**Control Group**	**2.0-4.0**	**2.5-6.0**

Figure S11 – The cells mechanical properties (the range of adhesion and Young's Modulus) measured by AFM.

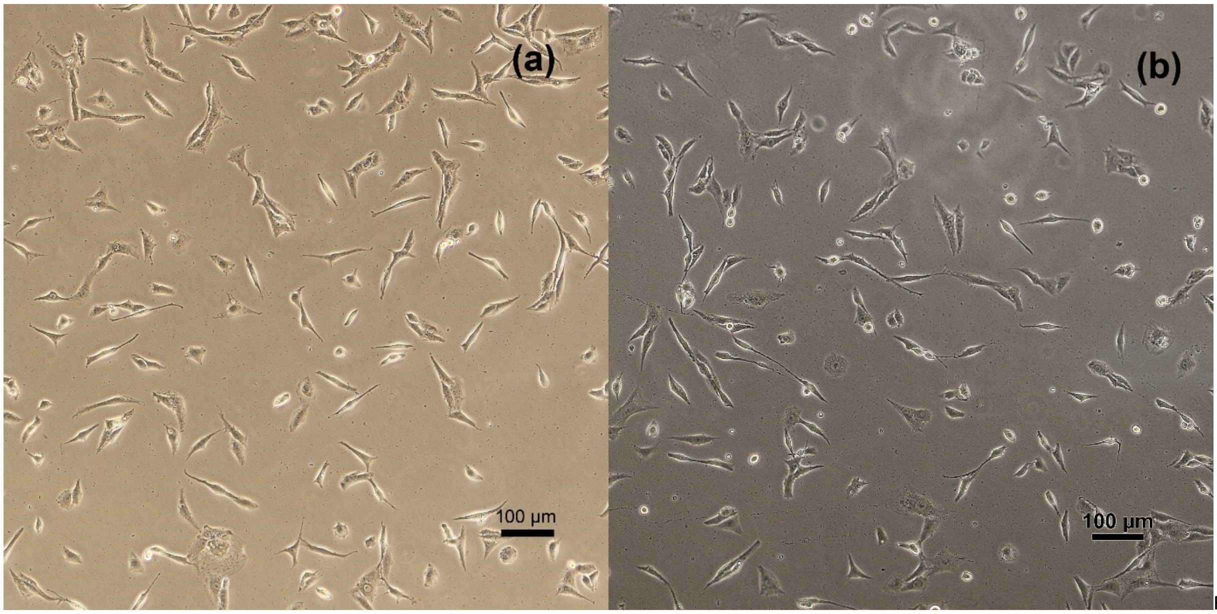

Figure S12 - The optical microscope images of bEnd.3 cells cultured in cover glass (a) and PPy substrate (b)

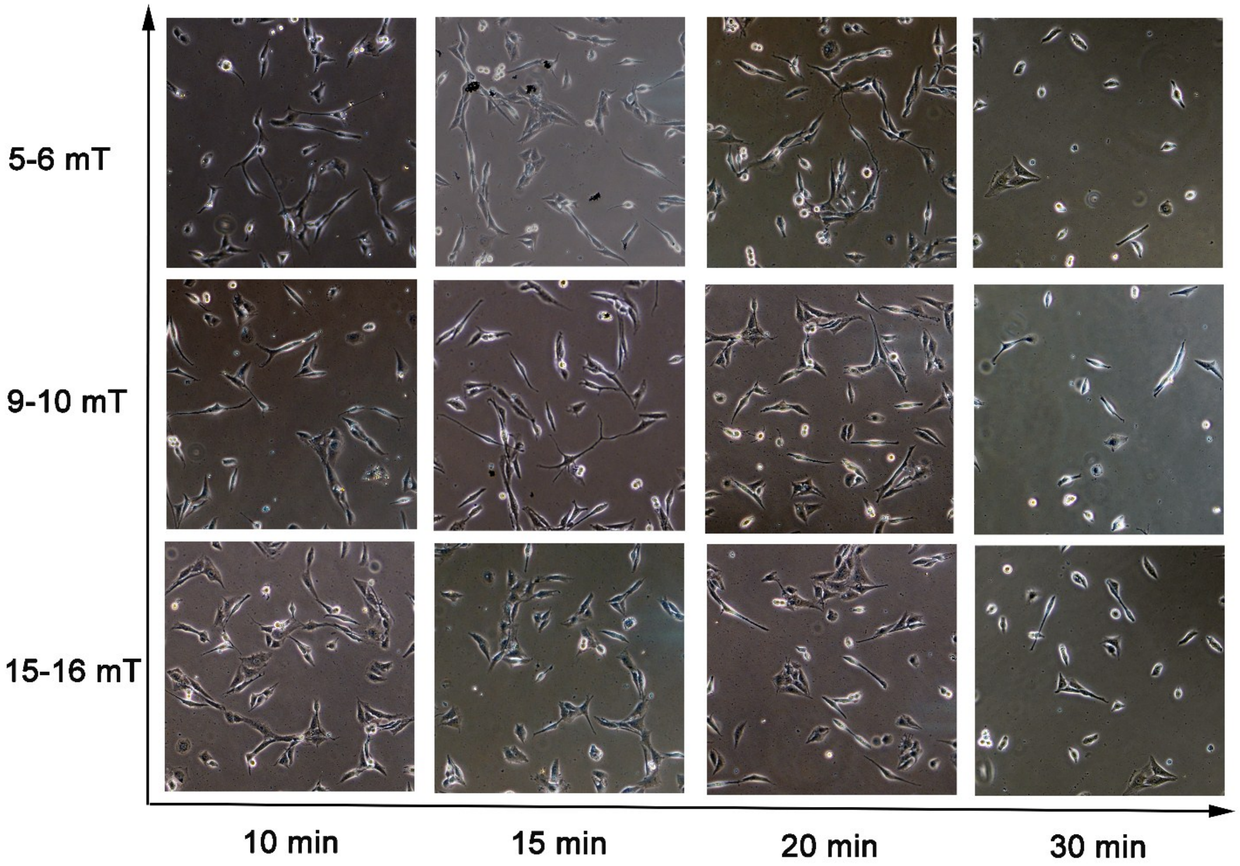

Figure S13 - The optical images of cells on polypyrrole substrate 2.3 after 10, 15, 20 and 30min magnetic stimulation with different magnetic intensities of 5-6 mT, 9 - 10 mT and 15 - 16 mT, respectively.

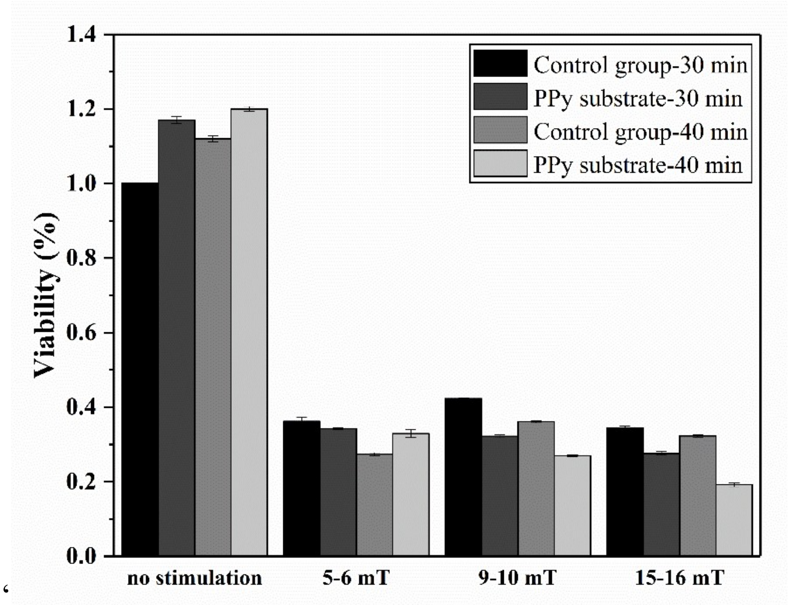

Figure S14 – Cell viability on polypyrrole substrate 2.3 or in the control group after 30 min and 40min magnetic stimulation with different magnetic intensities: 5 - 6 mT, 9 - 10 mT and 15 - 16 mT. The error bars represent the standard deviation.

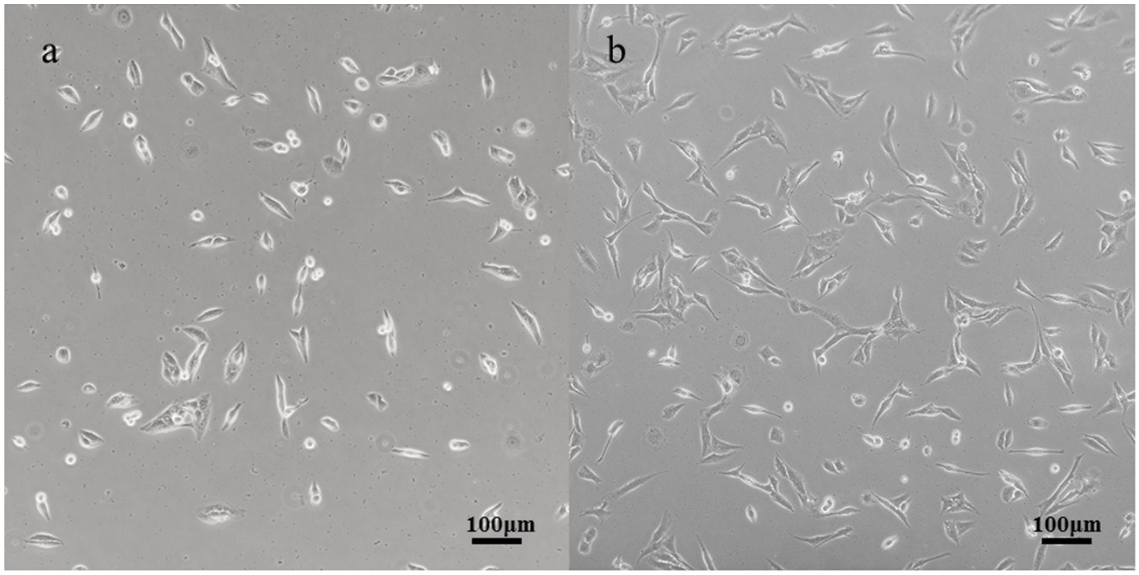

Figure S15 - The optical images of cells with low (a) and high (b) cell density on polypyrrole 2.3 substrate after 20 min magnetic stimulation with magnetic intensities of 9-10 mT; Scale bars: 100 μm.

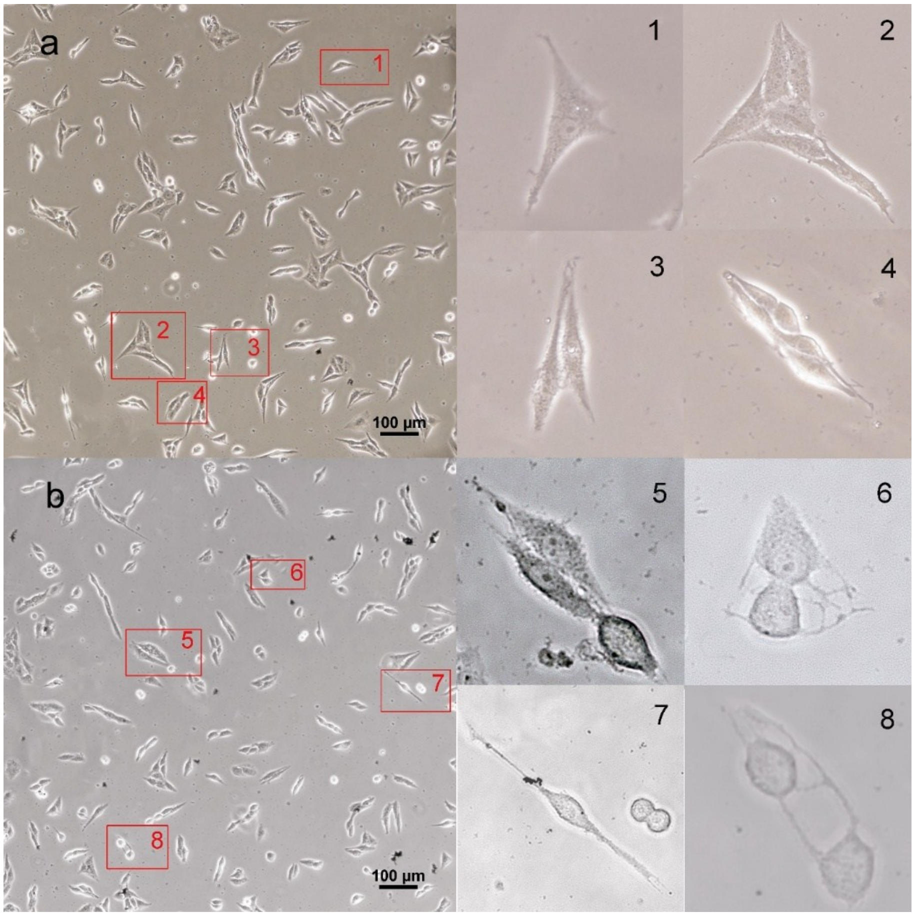

Figure S16 - The optical images of cells on polypyrrole substrate 2.3 before (a) and after (b) 20 min magnetic stimulation with magnetic intensities of 9 - 10 mT; Scale bars: 100 µm. (1-4) The magnifying images of cells in (a); (5-8) The magnifying images of cells in (b).

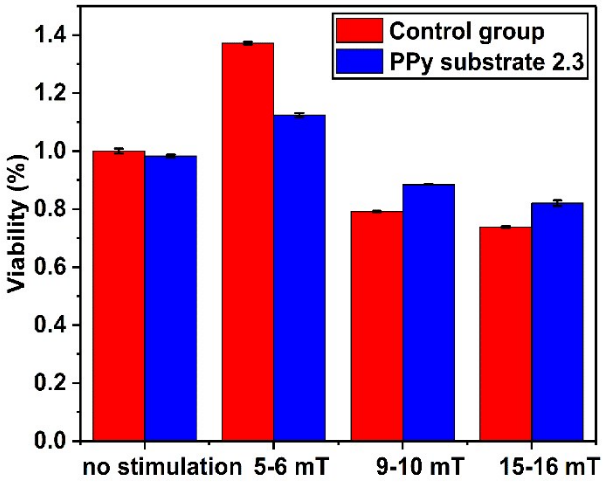

Figure S17 – Cell viability on polypyrrole substrate 2.3 or in the control group after 20 min magnetic stimulation with different magnetic intensities: 5 - 6 mT, 9 - 10 mT and 15 - 16 mT. Error bars represent the standard deviation.
